# PD226 Integration Of Patient And Clinician Insights Into The European Union Joint Scientific Consultations And Joint Clinical Assessments

**DOI:** 10.1017/S0266462324004458

**Published:** 2025-01-07

**Authors:** Elaine Julian, Robin Doeswijk, Rosa Giuliani, Wim Goettsch, Francine Brinkhuis, Bernhard J. Woermann, Mira Pavlovic, Antonella Cardone, Heiner C. Bucher, Begoña Pérez-Valderrama, Renato Bernardini, Walter Van Dyck, Maureen Rutten-van Moelken, Fabrizio Gianfrate, Oriol Solà-Morales, Mondher Toumi, Daniel Widmer, Tomas Salmonson, Jörg Ruof

## Abstract

**Introduction:**

To summarize insights generated during the preceding four conventions of the European Access Academy (EAA) regarding the interface of patient organizations and medical societies with the evolving European Union (EU) health technology assessment (HTA) process.

**Methods:**

In 2022 and 2023 four EAA conventions were held on the EU HTA regulation, focusing on: (i) its relevance for beating cancer; (ii) stakeholder involvement; (iii) recommended preparatory steps to ensure its successful implementation; and (iv) the role of hematology and oncology as a pacemaker for the EU HTA process. Here we summarize insights generated at the four EAA conventions about the integration of patient and clinician insights in the evolving EU HTA process, including joint scientific consultations (JSC) and joint clinical assessments (JCA).

**Results:**

Throughout the conventions it became clear that the interface of patient associations and clinical societies with the EU HTA process is key for successful implementation of the regulation. All involved stakeholders rely on the principles of evidence-based medicine (EBM), including best internal and external evidence, patient values and expectations, and clinical experience. It was agreed that patient and clinician perspectives on the assessments are needed to balance the technical analysis of best external evidence. While patient input is rather well defined, when and how input from clinical societies is best incorporated during the process remains unclear.

**Conclusions:**

As stipulated by the EBM triad, systematic involvement of patients and clinicians throughout both JSC and JCA is key to ensuring best outcomes for patients and society as a whole, in line with the objectives of the EU HTA regulation.

